# Production of dengue 2 envelope domain III in plant using CPMV - based vector system

**DOI:** 10.1186/1753-6561-8-S4-P80

**Published:** 2014-10-01

**Authors:** Lívia Érika Carlos Marques, Bruno Bezerra da Silva, Ilana Carneiro Lisboa Magalhães, Márcia Maria Marques Mendes, Lia Magalhães de Almeida, Maria Izabel Florindo Guedes

**Affiliations:** 1 Universidade Estadual Do Ceará, Fortaleza, Brazil

## Background

The envelope protein (E protein) of dengue virus is responsible for binding the virus to the host cell. This protein is considered an important immunogen for neutralization of the virus, the only able to induce the production of neutralizing antibodies [1]. The aim of this study was to use the Cowpea Mosaic Virus (CPMV) as a vector to express, on cowpea plants, *Vigna unguiculata *L. (Walp) the gene fragment that encodes for domain III of the E protein of dengue 2 virus.

## Methods

The cloning of inserts in non commercial plasmid (NCP) specific sites containing the RNA-2 of CPMV, its subsequent introduction into competent cells of *Escherichia coli *(DH10B) and the purification of these plasmids from the transformed cells was made through conventional molecular biology techniques. The chimerical virus was inoculated on cowpea plants, and the symptomatic leaves were processed for further purification of the recombinant protein. The protein was purified by a precipitation process described by Florindo *et al*. (2002) [2].

## Results and conclusions

The SDS-PAGE electrophoretic profile of EDIII protein revealed the migration of a protein fraction having a molecular mass between 45 and 66 kDa which was then recognized by specific anti-dengue antibodies by immunoblotting assay. The study shows that this expression system based on CPMV allows the production of recombinant protein maintaining its antigenic characteristics and demonstrates the potential of this platform for the transient production of proteins in plants.
